# An Artificial Intelligence-Based Fuzzy Logic System for Periodontitis Risk Assessment in Patients with Type 2 Diabetes Mellitus

**DOI:** 10.3390/bioengineering12030211

**Published:** 2025-02-20

**Authors:** Ioana Scrobota, Gilda Mihaela Iova, Olivia Andreea Marcu, Liliana Sachelarie, Siviu Vlad, Ioana Monica Duncea, Florin Blaga

**Affiliations:** 1 Department of Dental Medicine, Faculty of Medicine and Pharmacy, University of Oradea, 10 1st Decembrie Street, 410073 Oradea, Romania; ioana_scrobota@uoradea.ro (I.S.); gilda_iova@uoradea.ro (G.M.I.); 2 Preclinics Department, Faculty of Medicine and Pharmacy, University of Oradea, 10 1st Decembrie Street, 410073 Oradea, Romania; omarcu@uoradea.ro; 3 Department of Preclinical Discipline, Faculty of Medicine, Apollonia University, 700511 Iasi, Romania; 4 Department of Surgical Specialties, Faculty of Medicine and Pharmacy, University of Oradea, 10 1st Decembrie Street, 410073 Oradea, Romania; silviu.vlad@didactic.uoradea.ro; 5 Prosthetic Dentistry and Dental Materials Department, Iuliu Hațieganu University of Medicine and Pharmacy, 32 Clinicilor Street, 400006 Cluj-Napoca, Romania; ioana.duncea@umfcluj.ro; 6 Industrial Engineering Department, Faculty of Management and Technological Engineering, University of Oradea, 1 Universității Street, 410087 Oradea, Romania; fblaga@uoradea.ro

**Keywords:** diabetes mellitus, periodontology, periodontal disease, risk assessment, fuzzy logic

## Abstract

Background: Since periodontitis prevalence has increased globally and there is a bidirectional relationship between periodontitis and diabetes mellitus (DM), new methods of preventing and screening involving DM biomarkers could impact periodontitis management. We aimed to develop a fuzzy system to estimate the risk of periodontitis in patients with DM. Methods: Body mass index (BMI), glycemia (G), total cholesterol (C), and triglyceride (T) measurements were collected from 87 patients diagnosed with DM. Oral examinations were performed, and the number of the periodontal pockets (nrPPs) was determined. A fuzzy system was developed: BMI and G as inputs resulted in Periodontitis Risk 1 (PRisk1) output; C and T as inputs resulted in Periodontitis Risk 2 (PRisk2) output. From PRisk1 and PRisk2, the cumulative periodontitis risk (PCRisk) was assessed. Linguistic terms and linguistic grades (very small, small, medium, big, and very big) were assigned to the numerical variables by using 25 different membership functions. PCRisk and nrPP values were statistically processed. Results: In our developed fuzzy system, BMI, G, C, and T as input data resulted in periodontitis risk estimation. PCRisk was correlated with nrPP: when PCRisk increased by 1.881 units, nrPP increased by 1 unit. The fuzzy logic-based system effectively estimated periodontitis risk in type 2 diabetes patients, showing a significant correlation with the number of periodontal pockets. These findings highlight its potential for early diagnosis and improved interdisciplinary care.

## 1. Introduction

Periodontal diseases encompass a range of inflammatory conditions affecting the gingiva, bone, and periodontal ligament. The most severe form is periodontitis, characterized by progressive destruction of the teeth’s supporting structures. While periodontal diseases include conditions such as gingivitis and periodontal abscesses, this study focuses explicitly on periodontitis due to its strong association with systemic diseases like diabetes mellitus (DM) [1].

Periodontitis, the advanced form, affects 9.1% of adolescents, 36.6% of adults, and 48.7% of elderly individuals [2,3]. Severe periodontitis is reported in 8.5% of cases, with peak prevalence at 40 years [4,5,6]. Its economic burden necessitates improved prevention strategies [7].

Periodontitis results from microbial dysbiosis and host response. The oral microbiome triggers inflammation, while systemic factors like diabetes mellitus (DM) exacerbate disease progression [8,9].

DM is a metabolic disorder characterized by hyperglycemia due to insulin dysfunction. By 2045, 783 million people are expected to be affected [10,11]. Type 2 DM (T2DM) represents 90–95% of cases, associated with obesity and lifestyle factors [12,13,14]. DM complications, including cardiovascular and immune dysfunctions, worsen periodontal inflammation [15].

A DM–periodontitis relationship was recognized in 1990 when periodontitis was classified as a diabetes complication [16,17]. Hyperglycemia impairs periodontal tissues, while periodontitis-induced inflammation worsens glycemic control [18,19,20]. DM accelerates periodontal disease progression via immune dysregulation, oxidative stress, and bone resorption [11]. Altered microbial diversity in diabetics shows either reduced bacterial richness or increased pathogenicity [21,22].

At a cellular level, DM affects periodontitis via vascular and immune changes. Hyperglycemia impairs macrophage function, alters neutrophils, and promotes proinflammatory cytokines (IL-1β, IL-6, TNF-α) [23,24,25]. Oxidative stress and epigenetic changes perpetuate inflammation and tissue damage [26,27]. Periodontitis also exacerbates glycemic dysregulation in non-diabetics [28,29,30]. The number of periodontal pockets (nrPPs) is a key clinical indicator of periodontal disease severity. Periodontal pockets form due to inflammation-induced attachment loss, allowing bacterial colonization and disease progression. An increased nrPP value is associated with worsening periodontal conditions and is commonly used to assess disease burden in diabetic patients [31,32,33].

Despite DM-periodontitis awareness, periodontal risk assessment is often overlooked in diabetic care [31,32,33,34]. Traditional diagnosis relies on clinical indices, but AI-based systems, particularly fuzzy logic, offer advanced predictive models [35,36]. Unlike Boolean logic, fuzzy logic assigns degrees of risk, improving disease stratification [37,38].

Obesity, a DM risk factor, influences periodontitis via inflammation [39,40,41]. Dyslipidemia, with elevated cholesterol (C) and triglycerides (Ts), disrupts lipid metabolism and increases inflammation [42,43]. Cholesterol affects insulin secretion, reinforcing DM–periodontitis links [44,45,46]. Recognizing these associations, the American Diabetes Association (ADA) recommends routine lipid profiling [47].

Further research highlights inflammatory markers in DM and periodontitis, including increased C-reactive protein, oxidative stress, and hyperactive neutrophils, contributing to insulin resistance [48,49,50,51]. Neutrophil overactivity increases pro-inflammatory pathways, linking hyperlipidemia with periodontitis severity [52,53,54].

Additionally, genetic and epigenetic studies suggest specific polymorphisms influence both DM and periodontitis susceptibility, affecting immune and metabolic pathways [55,56,57,58].

Proteomic research identified biomarkers such as miR-96–5p, miR-7–5p, and growth differentiation factor-15 in DM, which may aid periodontitis screening [59,60,61,62]. Lipid alterations in DM impact immune function, with adiponectin, ferritin, and IL-2RA implicated in periodontitis progression [63,64,65,66]. Genetic predisposition, systemic inflammation, and metabolic dysregulation underscore the need for an integrated risk assessment approach [67,68,69,70].

Given the interrelated mechanisms of DM, obesity, and dyslipidemia in periodontal disease progression, this study develops a fuzzy logic-based system to assess periodontitis risk in T2DM patients. Using BMI, glycemia (G), total cholesterol (C), and triglycerides (Ts) as inputs, the system calculates a cumulative periodontal disease risk score (PCRisk), offering potential improvements in early diagnosis and preventive strategies [71,72,73,74].

The study aimed to develop and propose a system for evaluating the risk of developing periodontitis in diabetic patients by analyzing diabetes-related biomarkers, including body mass index (BMI), glycemia, total cholesterol (C), and triglycerides (Ts), using fuzzy logic, an artificial intelligence technique [44].

## 2. Materials and Methods

### 2.1. Participants

Of the 300 patients who visited Dr. Iova Gilda’s dental office between July 2018 and June 2019 and between April 2023 and September 2024, 87 patients over 18 consented to participate in the study. Of the total number of patients evaluated, 87 were selected after applying the inclusion criteria, which assumed a diagnosis of type 2 diabetes mellitus confirmed by the attending physician.

Diabetes mellitus is a significant factor involved in the pathogenesis of periodontitis, and smoking is one of the most important behavioral factors that aggravate this pathology. Even in the absence of other risk factors, the effects of smoking on periodontal tissues are well documented [58]. Studies show that smoking associated with diabetes generates a more pronounced synergistic effect than that observed in smokers without diabetes or in non-smoking diabetic individuals [59]. Therefore, smoking was an exclusion criterion from this study. Also excluded were patients with alcohol-related diseases, those diagnosed with other systemic diseases or conditions (such as autoimmune, rheumatological diseases, or active neoplasms), and those taking drug treatments that could influence periodontal health, such as corticosteroids or immunosuppressants. Patients who had received previous periodontal treatments were also excluded to eliminate variables that could influence the results.

The diagnosis of diabetes mellitus was established according to the American Diabetes Association (ADA) criteria, which include glycosylated hemoglobin values ≥ 6.5% obtained in two measurements, fasting blood glucose ≥ 126 mg/dL in two tests, or a result ≥ 200 mg/dL in the oral glucose tolerance test. The participants’ medical histories were documented, along with data on physical and biochemical parameters. The study was approved by the Ethics Committee of the Faculty of Medicine and Pharmacy, University of Oradea (nr. 5/15 June 2018). All participants were informed about the right to withdraw from the study at any time, in accordance with the ethical standards of the Declaration of Helsinki, revised in 2013. Participants agreed and signed the informed consent.

### 2.2. Biomarkers and Dento-Periodontal Examination

Body mass index (BMI) (kg/m^2^) and blood biochemistry tests, including glycemia (G) as indicated by fasting plasma glucose levels (mg/dL), total cholesterol (C) (mg/dL), and triglycerides (T) (mg/dL), were collected from patients’ personal records within 5 days of their visit to the general physician.

For each biomarker, values were collected from the patients’ medical records, and measurements were performed during routine check-ups with the attending physician. BMI was calculated as the ratio of body weight (in kilograms) to the square of height (in meters) [73]. Blood glucose was determined based on fasting plasma glucose values according to the criteria of the American Diabetes Association [71]. Total cholesterol and triglycerides were measured using standard clinical biochemistry assays commonly used to assess lipid profiles [57].

A complete oral examination was performed. The dental-periodontal examination included the evaluation of the Loe and Silness plaque index, missing teeth, bleeding on probing, clinical attachment level, and periodontal pockets. Periodontal probing depth was performed using a Wiliams probe placed into the pocket along the direction of the tooth root [60]. The probing depth was measured in six different sites of all remaining teeth in a subject: mesial, central, and distal for both the buccal and lingual sides of the tooth. Periodontitis was diagnosed according to the 2017 Classification of Periodontal and Peri-Implant Diseases and Conditions of The American Academy of Periodontology and the European Federation [61].

### 2.3. Development of the Fuzzy Procedure for Calculating the Cumulative (General) Risk Indicator for Periodontitis (PCRisk)

The decision support systems based on fuzzy were developed in Fuzzy Logic Toolbox—MATLAB following the steps indicated in Table 1.

PCRisk estimation was based on a two-level procedure.

The first level consisted of determining Periodontitis Risk 1 (PRisk1) using BMI and G criteria and calculating Periodontitis Risk 2 (PRisk2) using C and T criteria. The second level represented the PCRisk assessment by applying PRisk1 and PRisk2 criteria.

#### 2.3.1. The Fuzzy System of Periodontitis Risk 1 (PRisk1) Estimation

**Figure 1:**
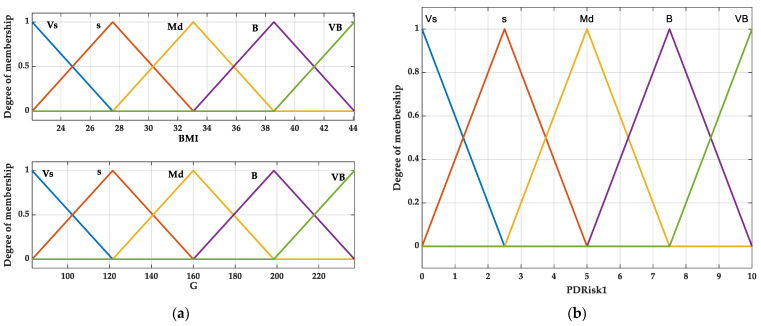
Fuzzy system of Periodontitis Risk 1 (PRisk1) estimation: (**a**) inputs; (**b**) output.

The impact of DM hyperglycemia on the development of periodontitis has been intensively studied, and the opinions are consensual. Recently, the theory of obesity indirectly influencing glycemia and directly affecting the periodontal tissues’ response to the oral microbiota via nonfunctional adipose tissue-dependent inflammation is of interest. Moreover, local inflammation due to periodontitis induces adipose tissue alteration, positioning DM, obesity, and periodontitis into a triangular self-maintenance relationship [62]. BMI and G were considered, therefore, as inputs (Figure 1a), and PRisk1 was considered as output (Figure 1b) in the decision support system.

The variation fields, LT, linguistic grades, and membership functions were associated with the inputs and outputs.

The dependence of the output on the inputs is described using inference “if-then” rules (the method of connecting different values of the membership functions).

25 inference rules were defined:

1. If (BMI is Vs) and (G is Vs), then (PRisk1 is Vs);

2. If (BMI is Vs) and (G is s), then (PRisk1 is Vs);

3. If (BMI is Vs) and (G is Md), then (PRisk1 is s);

…………………………………………………………

11. If (BMI is Md) and (G is Vs), then (PRisk1 is s);

12. If (BMI is Md) and (G is s), then (PRisk1 is Md);

13. If (BMI is Md) and (G is Md), then (PRisk1 is Md);

14. If (BMI is Md) and (G is B), then (PRisk1 is B);

15. If (BMI is Md) and (G is VB), then (PRisk1 is B);

………………………………………………………..

22. If (BMI is VB) and (G is s), then (PRisk1 is B);

23. If (BMI is VB) and (G is Md), then (PRisk1 is B);

24. If (BMI is VB) and (G is B), then (PRisk1 is VB);

25. If (BMI is VB) and (G is VB), then (PRisk1 is VB).

**Figure 2:**
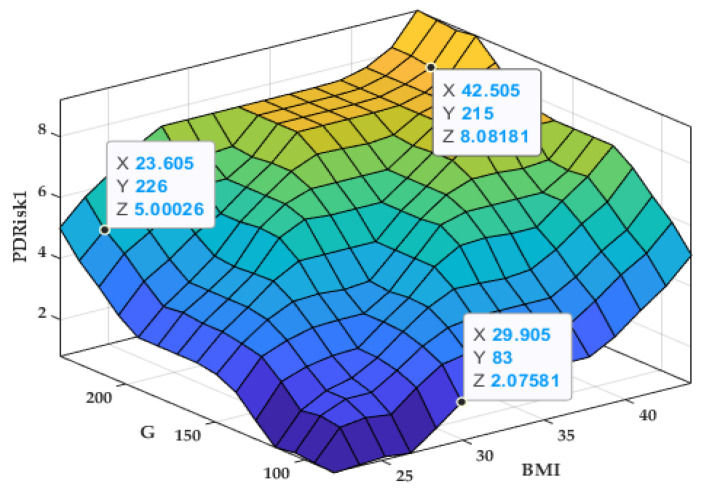
Variation surface PRisk1 = f (BMI, G).

The variation surface of the output PRisk1 depending on the inputs BMI and G is presented in Figure 2.

The variation surface in Figure 2 indicates that as both body mass index (BMI) and glycemia (G) increase, the estimated periodontal disease risk (PRisk1) also rises, suggesting a direct proportional relationship between these biomarkers and the risk level.

#### 2.3.2. The Fuzzy System of Periodontitis Risk 2 (PRisk2) Estimation

Dyslipidemia is essential to periodontitis progression and recent development [63,64,65]. T demonstrated a positive relation with the number of periodontal pockets in a study on 10,590 healthy subjects; otherwise, subjects in military service were screened for cardiovascular diseases [66]. At the same time, other authors found that both T and G in hyperlipidemic patients are associated with higher values of the periodontal parameters [67].

**Figure 3:**
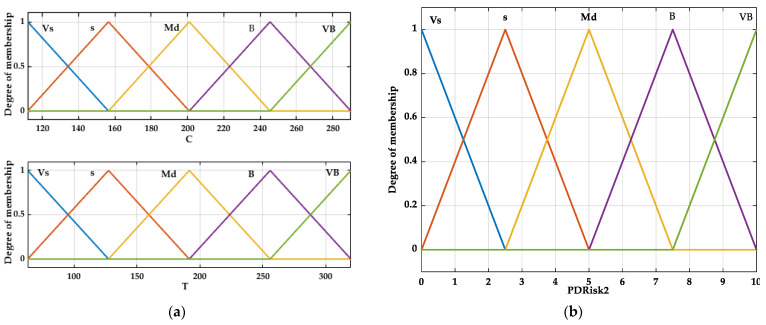
Fuzzy system of Periodontitis Risk 2 (PRisk2) estimation: (**a**) inputs; (**b**) output.

C and T were considered as inputs (Figure 3a), and PRisk2 was considered as output (Figure 3b) in the decision support system.

The variation fields, LT, linguistic grades, and membership functions were associated with the inputs and outputs.

The dependence of the output on the inputs is described using inference “if-then” rules (the method of connecting different values of the membership functions).

25 inference rules were defined:

1. If (C is Vs) and (T is Vs), then (PRisk2 is Vs);

2. If (C is Vs) and (T is s), then (PRisk2 is Vs);

3. If (C is Vs) and (T is Md), then (PRisk2 is s);

……………………………………………………….

11. If (C is Md) and (T is Vs), then (PRisk2 is s);

12. If (C is Md) and (T is s), then (PRisk2 is Md);

13. If (C is Md) and (T is Md), then (PRisk2 is Md);

14. If (C is Md) and (T is B), then (PRisk2 is B);

15. If (C is Md) and (T is VB), then (PRisk2 is B);

………………………………………………………

22. If (C is VB) and (T is s), then (PRisk2 is B);

23. If (C is VB) and (T is Md), then (PRisk2 is B);

24. If (C is VB) and (T is B), then (PRisk2 is VB);

25. If (C is VB) and (T is VB), then (PRisk2 is VB).

**Figure 4:**
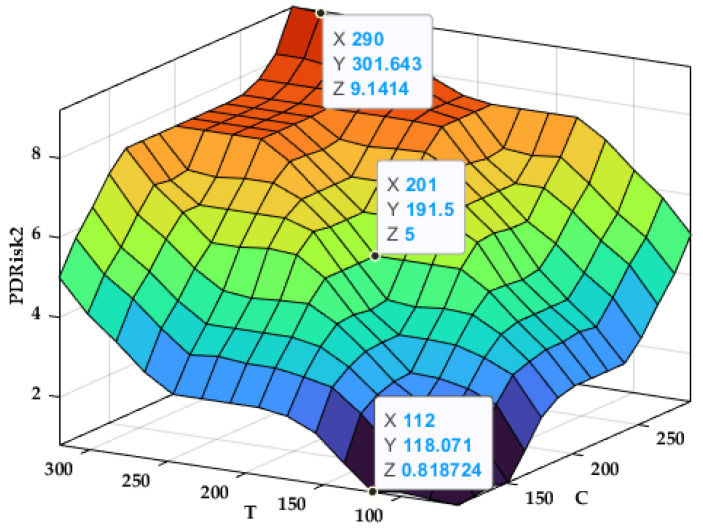
Variation surface PRisk2 = f (C, T).

The variation surface of the output Prisk2 depending on the inputs C and T is presented in Figure 4.

The variation surface in Figure 4 shows that as total cholesterol (C) and triglycerides (Ts) increase, the estimated periodontal disease risk (Prisk2) also rises, demonstrating their combined impact on the risk level.

#### 2.3.3. The Fuzzy System of Cumulative Periodontitis Risk (PCRisk) Estimation

**Figure 5:**
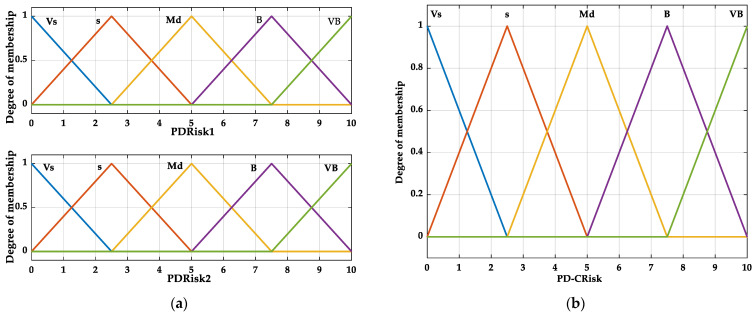
Fuzzy system for cumulative periodontitis risk (PCRisk) estimation: (**a**) inputs; (**b**) output.

Since the association of DM with dyslipidemia could be a risk indicator for periodontitis [4], PRisk1 and PRisk2 were considered as inputs (Figure 5a), and PCRisk was considered as output (Figure 5b) in the decision support system.

In addition to the medical justification of associating BMI, G, C, and T, respectively, in developing a fuzzy system and then assessing the cumulative risk, the necessity of using fewer inputs is also based on the fact that the system generates a large number of rules that reduce its efficacy by being time-consuming [68].

The variation fields, LT, linguistic grades, and membership functions were associated with the inputs and outputs.

The dependence of the output on the inputs is described using inference “if-then” rules (the method of connecting different values of the membership functions).

25 inference rules were defined:

1. If (PRisk1 is Vs) and (PRisk2 is Vs), then (PCRisk is Vs);

2. If (PRisk1 is Vs) and (PRisk2 is s), then (PCRisk is Vs);

3. If (PRisk1 is Vs) and (PRisk2 is Md), then (PCRisk is s);

…………………………………………………………………………

11. If (PRisk1 is Md) and (PRisk2 is Vs), then (PCRisk is s);

12. If (PRisk1 is Md) and (PRisk2 is s), then (PCRisk is Md);

13. If (PRisk1 is Md) and (PRisk2 is Md), then (PCRisk is Md);

14. If (PRisk1 is Md) and (PRisk2 is B), then (PCRisk is B);

15. If (PRisk1 is Md) and (PRisk2 is VB), then (PCRisk is B);

…………………………………………………………………………

22. If (PRisk1 is VB) and (PRisk2 is s), then (PCRisk is B);

23. If (PRisk1 is VB) and (PRisk2 is Md), then (PCRisk is B);

24. If (PRisk1 is VB) and (PRisk2 is B), then (PCRisk is VB);

25. If (PRisk1 is VB) and (PRisk2 is VB), then (PCRisk is VB).

**Figure 6:**
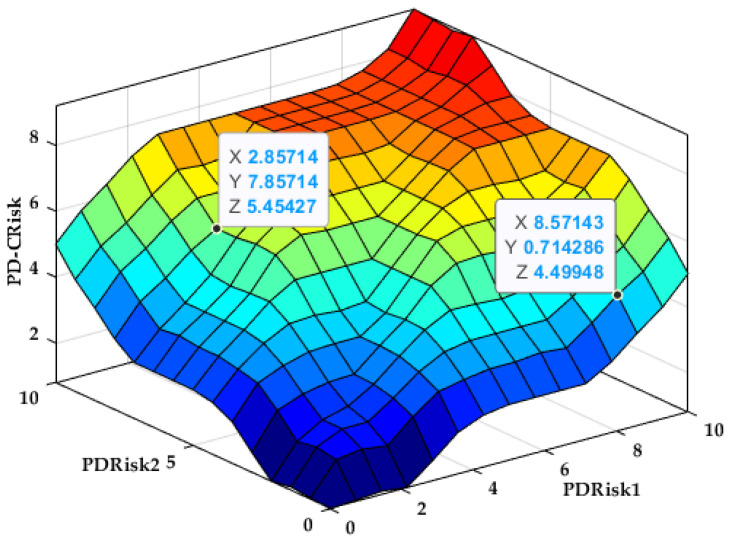
Variation surface PCRisk = f (PRisk1, PRisk2).

The variation surface of the output PCRisk depending on the inputs PRisk1 and PRisk2 is presented in Figure 6.

The variation surface in Figure 6 illustrates how the estimated overall periodontitis risk (PCRisk) is influenced by the combined inputs PRisk1 and PRisk2, highlighting their synergistic effect on the final risk assessment.

## 3. Results

**Figure 7:**
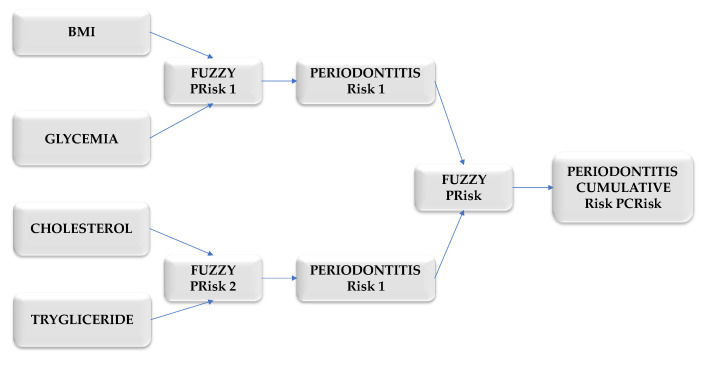
The procedure for calculating the cumulative (general) risk indicator for periodontal disease (PCRisk).

Figure 7 shows the procedure for calculating the cumulative (general) risk indicator for periodontal disease (PCRisk).

The cumulative (general) risk indicator for periodontal disease, PCRisk, was determined using the fuzzy procedure.

Each specific parameter was introduced in a different interval from all patients, delimited by the minimum and maximum values of the registered parameters. The minimum and maximum values of each biomarker (Table 2) were used to develop the fuzzy procedures.

Following the clinical examination of the patient’s periodontal pockets, the nrPPs were registered (Table S1).

Descriptive statistics of PCRisk and nrPP were performed. Half of the patients with the lowest fuzzy estimated PCRisk had values between 2.41 and 5.6475, and the other half had higher values between 5.6475 and 7.5. Half of the patients presented between 0 to 10 periodontal pockets, while the other half had 10 to 20 (Table 3).

In Table 4, it is observed that the regression model of nrPP is statistically significant.

The regression model is linear and expresses a directly proportional relationship between the fuzzy estimated periodontal risk and the number of periodontal pockets depicted. When PCRisk increased by 1.881 units, nrPP increased by 1 unit.

nrPP = 0.399 + 1.881 × PCRisk (Table 5).

## 4. Discussion

The present study developed a fuzzy system using BMI, G, C, and T to estimate the risk of periodontitis. A direct proportional relationship was found between the fuzzy-estimated periodontal risk and the number of periodontal pockets (nrPP) in our patients, indicating that an increased PCRisk value corresponding to a higher number of periodontal pockets.

Our results align with previous studies confirming the bidirectional relationship between DM and periodontitis [37]. In the early stages of periodontal deterioration, patients often overlook symptoms, leading to disease progression over time [69]. Multiple factors influence periodontitis progression, impacting treatment outcomes and patient quality of life [70]. Since DM affects both the prevalence and severity of periodontitis [37], assessing DM-related biomarkers may improve early diagnosis and disease management.

The fuzzy multi-criteria decision support system developed in this research processed user-entered data (inputs) to provide an estimated risk output. The biomarkers selected for the fuzzy system—G, BMI, C, and T—were chosen based on their strong association with DM and periodontitis risk.

Glycemia (G) was included as it plays a key role in DM diagnosis and management. Hyperglycemia contributes to periodontitis through genetic, inflammatory, and oxidative stress pathways that disrupt immune responses, activate osteoclasts, and alter polymorphonuclear leukocyte activity, ultimately leading to periodontitis [72]. Several methods can assess glycemia, including fasting plasma glucose levels, the 2 h oral glucose tolerance test, and glycated hemoglobin, all effective for DM diagnosis [71].

BMI was selected as it serves as an indicator of obesity, a known risk factor for both DM and periodontitis. BMI exceeding 25 kg/m^2^ is associated with metabolic dysregulation that alters immune responses and promotes systemic inflammation [73,74]. Obesity contributes to periodontitis through increased levels of proinflammatory cytokines, hypersecretion of adipokines, and enhanced macrophage infiltration, all of which worsen periodontal inflammation [75,76].

Dyslipidemia is a well-established comorbidity of DM and has also been linked to periodontitis [77]. Diabetic individuals are predisposed to hypercholesterolemia [78], elevated cholesterol, and triglycerides have been shown to predict disease outcomes in diabetic patients [79]. Dyslipidemia contributes to periodontitis through systemic inflammation, immune dysregulation, and impaired healing mechanisms [63,65]. Recent studies indicate that hyperlipidemia alters leukocyte activity, increases macrophage differentiation, and enhances the production of reactive oxygen species, further promoting periodontal damage [80,81].

Several biomarkers have been studied as potential indicators for DM onset and progression [82]. Genetic research has identified hundreds of genetic risk factors contributing to DM development, with future advancements in gene therapy and epigenetic modifications potentially improving disease management [83,84,85]. Additionally, proteomic biomarkers, including micro(mi)RNAs such as miR-96–5p, miR-7–5p, and miR-132, have been investigated for their role in DM diagnostics and risk assessment [86]. Other molecular markers have been associated with DM progression, including aminoadipic acid, homocitrulline, and growth differentiation factor 15 [82,87,88]. While advanced diagnostic tools such as genetic and microbiological tests could improve periodontitis screening, they require specialized personnel, equipment, and financial resources, limiting their widespread use [89]. The biomarkers assessed in this study (G, BMI, C, and T) are routinely measured during medical check-ups and can be self-monitored by patients at home [90,91,92]. A future fuzzy logic-based interface that integrates self-collected biomarker data could simplify periodontitis risk assessment for diabetic patients, making early detection more accessible.

One of the main advantages of fuzzy logic is its ability to translate human-like reasoning into decision-making models. By using linguistic terms (LTs) such as very small (Vs), small (S), medium (M), big (B), and very big (VB), the fuzzy system enables an intuitive interpretation of data [93]. Unlike traditional classification models, fuzzy logic accommodates the uncertainty inherent in medical diagnosis by applying “if-then” rules, enhancing its applicability in risk assessment [94].

Despite its advantages, fuzzy logic models’ accuracy depends on expert knowledge in defining input parameters, membership functions, and inference rules [95]. Alternative approaches, such as random forest and logistic regression models, have also been studied for periodontitis risk assessment in DM patients [89,94,95,96,97,98,99,100]. Furthermore, machine learning models, including AutoML H2O eXtreme Gradient Boosting, have been used to predict metabolic syndrome by incorporating periodontitis stage, cardiovascular risk factors, and health-related quality of life indicators [101].

Artificial intelligence techniques, particularly fuzzy logic-based models, have been explored for periodontal disease diagnosis and risk assessment. Allahverdi et al. [102] developed a fuzzy expert system for diagnosing periodontal disease, demonstrating its ability to assist clinicians by integrating multiple diagnostic criteria. Similarly, Nour et al. [103] proposed an AI-driven model to enhance dental healthcare systems, emphasizing the role of AI in improving diagnostic accuracy and clinical decision-making. These findings support the potential of our fuzzy logic-based approach in periodontal disease risk estimation for diabetic patients, reinforcing its applicability in clinical practice.

Artificial intelligence (AI) has been increasingly applied in medical risk assessment, mainly through fuzzy logic-based models. Marsh and Khuman [104] developed a fuzzy logic risk assessment system for type 2 diabetes, demonstrating how AI-driven approaches can integrate multiple clinical parameters for improved predictive accuracy. Similarly, our study utilizes fuzzy logic to estimate periodontal disease risk in diabetic patients, further supporting the role of AI in enhancing disease prediction and personalized healthcare management.

Fuzzy logic has been successfully applied to risk assessment in various pathologies, including DM [104], colorectal cancer [105], neurosurgical outcomes [106], and oral cancer. This is the first study to propose a fuzzy logic-based decision support system for periodontitis risk estimation in DM patients. Such a system could be valuable for improving periodontitis screening and management, particularly in primary care settings.

Implementing a fuzzy logic-based risk assessment model could enhance the collaboration between general physicians and dentists, leading to earlier referrals and improved patient outcomes. Integrating systemic and oral health assessments may improve overall health and quality of life for diabetic patients.

However, this study has limitations, including a relatively small sample size and a focus on adult and elderly diabetic patients, which may limit its applicability to younger or prediabetic populations. Despite these constraints, the proposed fuzzy logic system provides a solid foundation for periodontitis risk estimation in DM patients, with potential for further refinement and expansion.

Future research should optimize the fuzzy logic model, incorporate additional clinical and behavioral parameters, and explore its integration with self-collected biomarker data for remote health monitoring. Such advancements could facilitate a more personalized and accessible approach to periodontitis risk assessment in diabetic patients.

## 5. Conclusions

This study highlights the effectiveness of a fuzzy logic-based system for estimating periodontitis risk in type 2 diabetes patients using accessible biomarkers (BMI, glycemia, cholesterol, and triglycerides). A significant direct correlation was identified between the fuzzy-estimated risk (PCRisk) and the number of periodontal pockets (nrPP), indicating that metabolic biomarkers are key in periodontal disease progression. These findings reinforce the potential of this system for improving early periodontitis diagnosis and facilitating interdisciplinary care between medical and dental professionals.

## Figures and Tables

**Table 1:** Well-defined steps in developing fuzzy logic-based decision support systems.

Step	Description
1. Defining criteria	Input data relevant to decision-making were identified.
2. Setting variation fields for inputs	The range of numerical input values, from minimum to maximum, was determined based on the biomarkers considered.
3. Allocating linguistic terms (LTs) for inputs	Numerical input values were categorized into linguistic terms such as very small (Vs), small (S), medium (Md), big (B), and very big (VB).
4. Assigning linguistic grades to inputs	Each linguistic term was assigned a grade to quantify its meaning in decision-making.
5. Establishing membership functions for inputs	Functions were created to associate linguistic terms with their corresponding numerical input values.
6. Defining output data	The output variables required for decision-making were specified.
7. Setting variation fields for outputs	The range of output values was defined, typically from 1 to 10.
8. Allocating linguistic terms for outputs	Output values were also categorized using linguistic terms such as Vs, S, Md, B, and VB.
9. Assigning linguistic grades to outputs	Similar to the inputs, linguistic grades were assigned to the output categories.
10. Establishing membership functions for outputs	Functions were developed to map linguistic terms to their respective numerical output values.
11. Defining the connection method for membership functions	The approach to combining and processing membership function values was decided to ensure accurate results.

**Table 2:** Minimum and maximum values of the considered biomarkers.

Biomarkers					
**Values**		**BMI (kg/m^2^)**	**G (mg/dL)**	**C (mg/dL)**	**T (mg/dL)**
	Min	22.03	83	112	63
	Max	44.08	237	290	320

**Table 3:** Descriptive statistics of PCRisk and nrPP.

Statistics	PCRisk	nrPP
Mean	5.4936	10.7297
Median	5.6475	10.0000
Std. deviation	1.30751	6.52197
Minimum	2.41	0.00
Maximum	7.50	20.00

**Table 4:** ANOVA analysis of PCRisk and nrPP.

Model	Sum of Squares	df	Mean Square	F	Sig.
Regression	217,650	1	217,650	5.799	0.021
Residual	1,313,647	35	37,533		
Total	1,531,297	36			

Dependent variable: nrPP; predictors: (constant), PCRisk.

**Table 5:** Regression model coefficients.

Model	Unstandardized Coefficients B	Std. Error	Standardized Coefficients Beta	t	Sig.
(Constant)	0.399	4.407		0.090	0.928
PCRisk	1.881	0.781	0.377	2.408	0.021

Dependent variable: nrPP.

## Data Availability

Data is available from the corresponding author upon reasonable request.

## Supplementary Materials

The following supporting information can be downloaded at: https://www.mdpi.com/article/10.3390/bioengineering12030211/s1, Table S1 Fuzzy estimated Periodontitis_CRisk and clinically determined nrPP in DM patients.
