# Supplementary material for: An Artificial Intelligence-Based Fuzzy Logic System for Periodontitis Risk Assessment in Patients with Type 2 Diabetes Mellitus
Source: Bioengineering (Basel). 2025 Feb 20;12(3):211. doi: 10.3390/bioengineering12030211 (PMC11939156; doi:10.3390/bioengineering12030211)
Supplement: Supplementary file 1 [file bioengineering-12-00211-s001.zip › bioengineering-3460070-supplementary.pdf]

Fuzzy estimated PCRisk and clinically determined nrPP in DM patients

Patients	Fuzzy estimated PCRisk	nrPP
1.	4.1159	4
2.	6.194	7
3.	4.5829	0
4.	5.6192	18
5.	3.6034	4
6.	6.4079	0
7.	5.6992	10
8.	6.3141	9
9.	6.4956	8
10.	7.1707	17
11.	7.2053	16
12.	6.0921	0
13.	6.0337	8
14.	4.104	5
15.	5.6475	12
16.	5.5616	7
17.	4.3522	0
18.	6.5367	4
19.	3.426	8
20.	4.6812	17
21.	7.4969	20
22.	4.9961	16
23.	3.2161	2
24.	5	13
25.	5.197	17
26.	3.7411	8
27.	2.4081	10
28.	4.839	20
29.	6.3303	18
30.	4.1728	13
31.	7.497	20
32.	5.5327	9
33.	6.4432	13
34.	6.3941	16
35.	6.9136	20
36.	5.9226	20
37.	7.3192	8
